# A Web-Based, Social Networking Physical Activity Intervention for Insufficiently Active Adults Delivered via Facebook App: Randomized Controlled Trial

**DOI:** 10.2196/jmir.4086

**Published:** 2015-07-13

**Authors:** Carol Maher, Monika Ferguson, Corneel Vandelanotte, Ron Plotnikoff, Ilse De Bourdeaudhuij, Samantha Thomas, Karen Nelson-Field, Tim Olds

**Affiliations:** ^1^ Alliance for Research in Exercise, Nutrition and Activity University of South Australia Adelaide Australia; ^2^ Institute for Health and Social Sciences Central Queensland University Adelaide Australia; ^3^ Priority Research Centre in Physical Activity and Nutrition University of Newcastle Newcastle Australia; ^4^ Department of Movement and Sports Sciences University of Ghent Ghent Belgium; ^5^ School of Health and Society University of Wollongong Wollongong Australia; ^6^ Ehrenberg-Bass Institute for Marketing Science University of South Australia Adelaide Australia

**Keywords:** social network, behavior change, intervention, Internet, physical activity

## Abstract

**Background:**

Online social networks offer considerable potential for delivery of socially influential health behavior change interventions.

**Objective:**

To determine the efficacy, engagement, and feasibility of an online social networking physical activity intervention with pedometers delivered via Facebook app.

**Methods:**

A total of 110 adults with a mean age of 35.6 years (SD 12.4) were recruited online in teams of 3 to 8 friends. Teams were randomly allocated to receive access to a 50-day online social networking physical activity intervention which included self-monitoring, social elements, and pedometers (“Active Team” Facebook app; n=51 individuals, 12 teams) or a wait-listed control condition (n=59 individuals, 13 teams). Assessments were undertaken online at baseline, 8 weeks, and 20 weeks. The primary outcome measure was self-reported weekly moderate-to-vigorous physical activity (MVPA). Secondary outcomes were weekly walking, vigorous physical activity time, moderate physical activity time, overall quality of life, and mental health quality of life. Analyses were undertaken using random-effects mixed modeling, accounting for potential clustering at the team level. Usage statistics were reported descriptively to determine engagement and feasibility.

**Results:**

At the 8-week follow-up, the intervention participants had significantly increased their total weekly MVPA by 135 minutes relative to the control group (*P*=.03), due primarily to increases in walking time (155 min/week increase relative to controls, *P*<.001). However, statistical differences between groups for total weekly MVPA and walking time were lost at the 20-week follow-up. There were no significant changes in vigorous physical activity, nor overall quality of life or mental health quality of life at either time point. High levels of engagement with the intervention, and particularly the self-monitoring features, were observed.

**Conclusions:**

An online, social networking physical activity intervention with pedometers can produce sizable short-term physical activity changes. Future work is needed to determine how to maintain behavior change in the longer term, how to reach at-need populations, and how to disseminate such interventions on a mass scale.

**Trial Registration:**

Australian New Zealand Clinical Trials Registry (ANZCTR): ACTRN12614000488606; https://www.anzctr.org.au/Trial/Registration/TrialReview.aspx?id=366239 (Archived by WebCite at http://www.webcitation.org/6ZVtu6TMz).

## Introduction

Physical inactivity is a leading modifiable cause of death and disease worldwide and causes as many deaths as smoking [1]. Just 30 minutes a day of moderate-intensity physical activity halves the risk of leading causes of morbidity and mortality, such as cardiovascular disease, type 2 diabetes, and obesity, and reduces the risk of breast and bowel cancer, depression, and anxiety [2]. Despite this, many people in developed countries are insufficiently active to achieve these benefits. For example, a recent nationally representative survey of 20,426 Australians found that 67% of adults self-reported that they got less than 30 minutes of physical activity a day [3]. Population-based interventions are needed to assist the general adult population to become more physically active.

Web-based physical activity interventions offer an opportunity to reach a large number of people at a relatively low cost. Systematic reviews and meta-analyses of Web-based physical activity interventions demonstrate they are effective in changing behavior [4,5], however, typically they have not been adopted by large numbers of users and appear to have difficulty sustaining user engagement over an extended period [4,5]. New intervention approaches capitalizing on recent technology trends, such as online social networks [6] and gamification, may assist in overcoming these issues. Our recent review of online social networks for delivery of health behavior interventions found fledgling, but promising, evidence of effectiveness [7].

Online social networks reportedly account for one-quarter of all time spent online [8,9], and appear to offer considerable potential for delivery of public health campaigns for several reasons. Like the Internet in general, they can reach very large audiences (eg, Facebook, the world’s largest social networking website, had 1.32 billion users each month as of June 2014 [10]). They also offer some key advantages over conventional online delivery, including that messages can be delivered via existing social contacts, which may be more influential than health messages delivered via traditional marketing strategies [11]. Furthermore, unlike traditional Web-based interventions [4], online social networks typically achieve high levels of user engagement and retention [12].

Another online trend that has emerged in recent years is gamification. Gamification refers to the application of video game elements, such as fun, challenges, competition, and rewards, in nongaming situations [13]. In the commercial sector, such techniques have reported to markedly increase engagement (eg, a software company reported an 8-fold increase in user engagement after introduction of gamification features) [14]. A recent systematic review of health behavior change interventions delivered using online social networks [7] found that compared to studies which did not incorporate gamification features, the one study that did—in the form of competition between users [15]—achieved substantially larger intervention effects and higher levels of user engagement.

To date, only a handful of studies have attempted to use existing popular online social network platforms, such as Facebook and Twitter, to intervene on physical activity. The most common approach has been the use of a Twitter feed or private Facebook groups to share content regarding physical activity and facilitate discussion between study participants [7]. In most cases, the online social network intervention has been provided as a component, complementing a more comprehensive intervention package, for example, involving access to a physical activity self-monitoring website with personalized feedback from a health professional [16]; provision of pedometer, digital scales, cooking equipment, and personalized feedback [17]; or access to a series of podcasts, advice from an expert moderator, and a calorie-counting app [18]. To our knowledge, only one previous study [15] has utilized a Facebook app (ie, software created by third party developers to function within the Facebook platform and access data in Facebook) to intervene on physical activity.

The primary objective of this study was to determine whether a team-based 50-day social networking physical activity intervention delivered via Facebook app and incorporating gamification features was effective in changing weekly moderate-to-vigorous physical activity (MVPA) in adults aged 18 to 65 years. The secondary objectives were (1) to determine whether the intervention impacted other physical activity (ie, weekly walking, vigorous physical activity, and moderate physical activity time) and quality of life (in particular, mental quality of life), (2) to determine usage, and (3) to examine the feasibility of the online intervention.

## Methods

### Overview

Ethical approval for this cluster randomized controlled trial (RCT) was obtained from the University of South Australia Human Research Ethics Committee, and the study was registered with the Australian and New Zealand Clinical Trials Registry, protocol number: ACTRN12614000488606. Data collection took place between September 2013 and July 2014. Participants provided informed consent online prior to commencing the study. The study was designed, and the manuscript prepared, following CONSORT guidelines [19].

### Intervention

**Figure 1:**
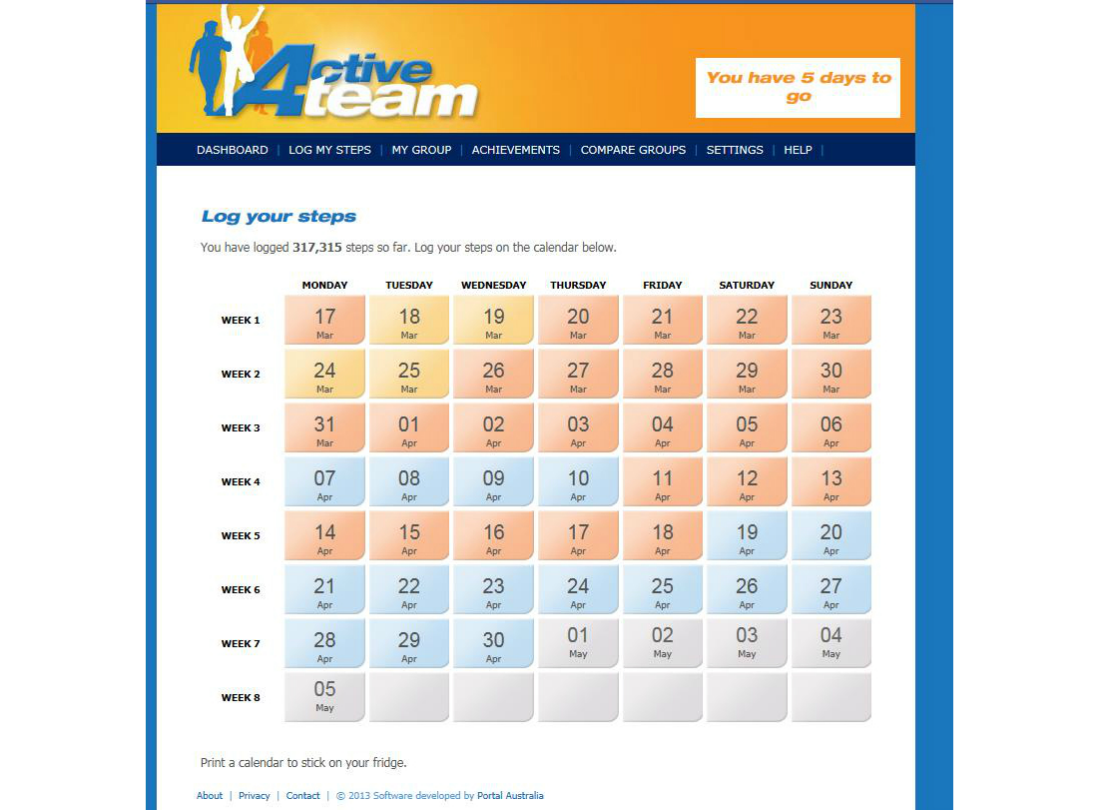
Active Team step-logging calendar.

**Figure 2:**
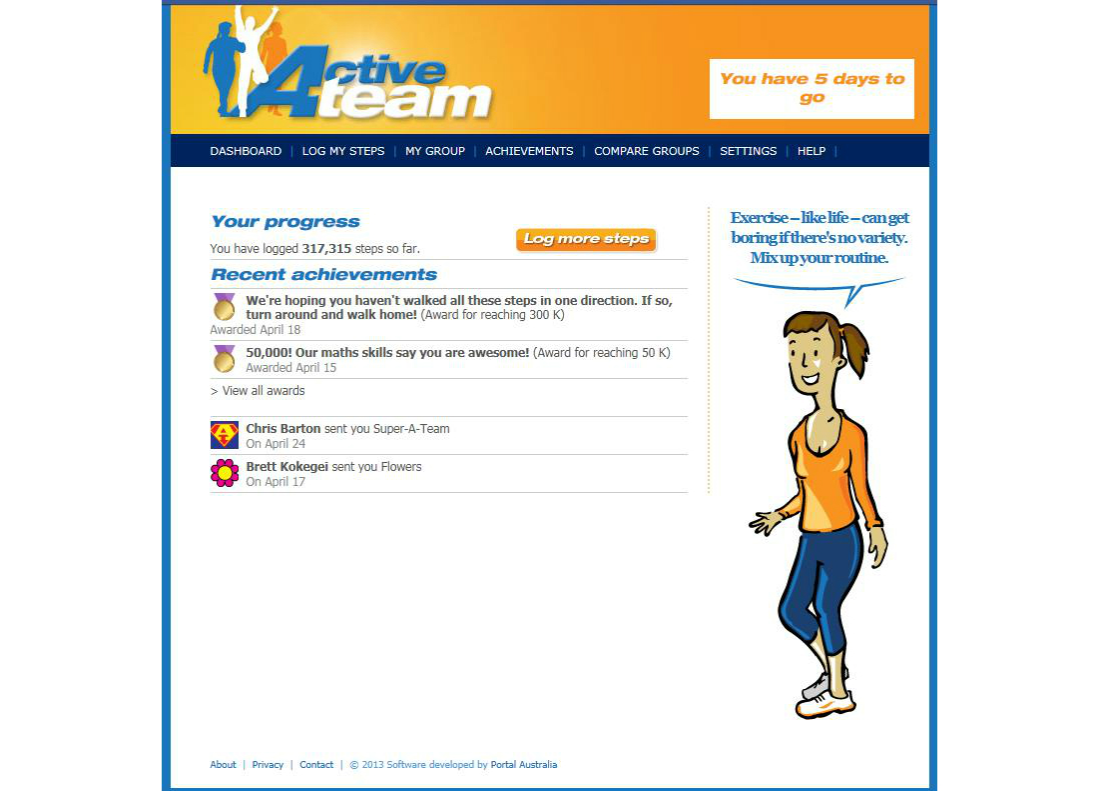
Active Team dashboard, showing step-logging progress, awards, and gifts.

Active Team is a new, free, 50-day team-based Facebook app, developed to assist adults to increase their physical activity levels. The content and features of the program were developed by a team at the University of South Australia led by Dr Carol Maher, following a series of interviews with 20 adults regarding the potential for developing a physical activity intervention delivered via online social networks (unpublished). Commercial software developers were engaged to produce the software platform, and extensive pilot-testing and usability testing was undertaken for the first version of the software [20]. Participants are provided with a pedometer, and encouraged to achieve 10,000 steps per day [21], working in teams of 3 to 8 existing Facebook friends. Active Team is designed to encourage friendly rivalry within friendship groups, offer peer encouragement and support, and be quick, social, and enjoyable to use. It includes a calendar to log daily step counts (steps can be logged up to 7 days in arrears) (see Figure 1); a dashboard showing step-logging progress, awards, and gifts (see Figure 2); a team tally board to allow users to monitor their own and their teammates’ progress; a team message board for team members to communicate with one another; daily tips for increasing physical activity; gamification features, such as awards for individual and team step-logging and step-count achievements; and the ability to send virtual gifts to teammates. Automated computer-tailored weekly emails are sent to participants summarizing their progress and encouraging continued participation. Apart from provision of a pedometer, the Active Team intervention approach was designed to be minimally resource intensive and, therefore, did not include provision of extensive instrumental support, expert moderation, or feedback from a health professional.

Following consideration of numerous behavior change theories, the theory of planned behavior [22,23] and fun theory [24] were selected to inform development of the content and features of Active Team. The theory of planned behavior posits that a person’s decision to perform a particular behavior is influenced by three factors: attitude, subjective norms, and perceived behavioral control [22,23]. Fun theory advocates that people will be more motivated to do routine activities if they are adapted to be fun [24]. The Active Team app attempts to address each of these factors by providing daily tips for physical activity, written by a comedian (theory of planned behavior—attitudes and perceived behavioral control; fun theory); use of teams for peer encouragement and support (theory of planned behavior—subjective norms; fun theory); and setting small achievable goals (ie, daily step count), which are recorded and contribute to a long-term/overall goal (500,000 steps) (theory of planned behavior—attitude and perceived behavioral control), unlockable awards, named by a comedian (fun theory), and the ability to send virtual gifts, such as a high five and a pink leotard (theory of planned behavior—subjective norms; fun theory).

### Participants and Procedures

**Figure 3:**
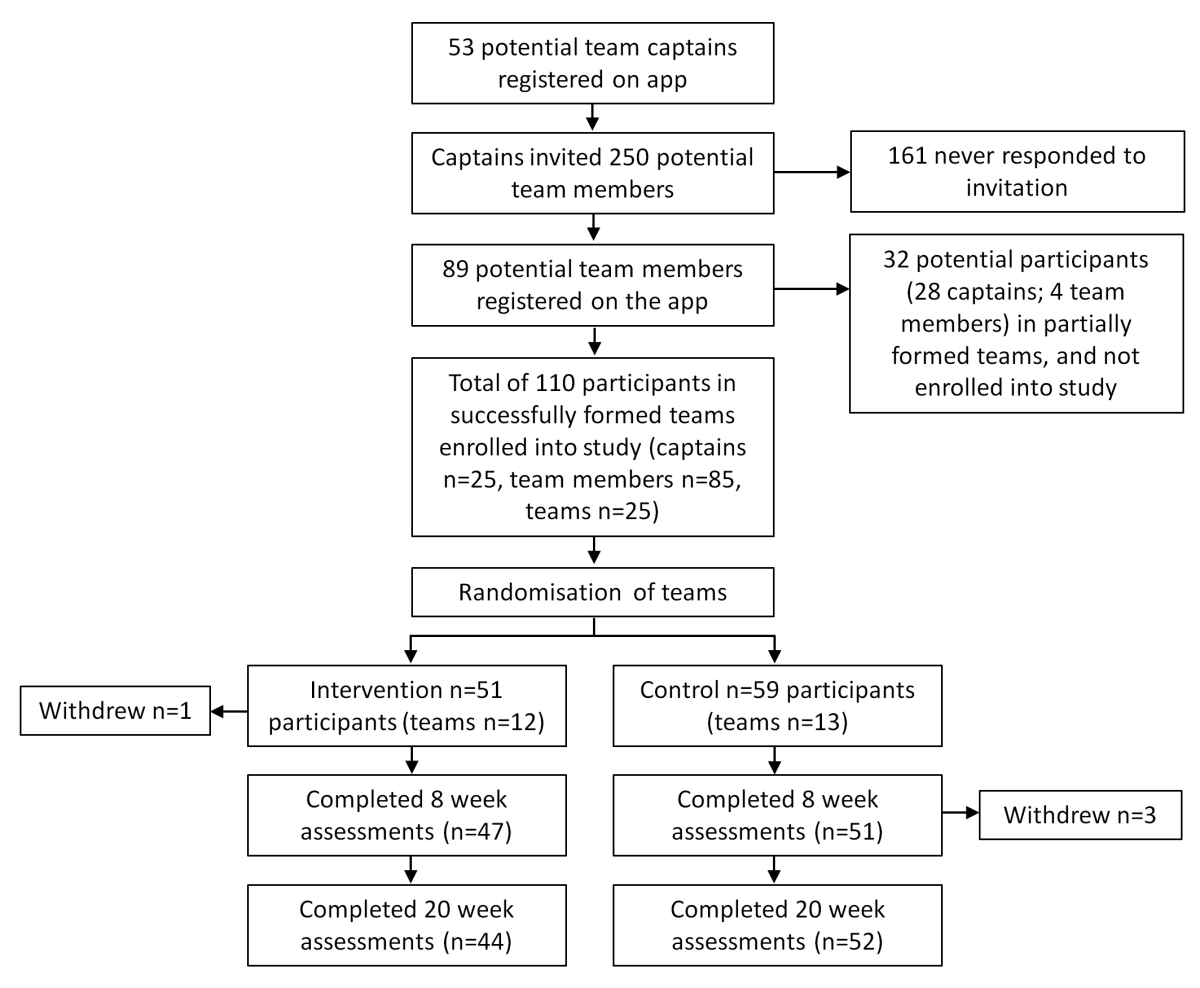
Overview of participant recruitment, assessment, and flow.

An overview of the randomized controlled trial methodology is shown in Figure 3. Participants were recruited through a Facebook advertising campaign, media stories in the local newspaper and television news bulletin, and distribution of flyers at the University of South Australia campuses. Participants were eligible if they met the following criteria: (1) were between the ages of 18 and 65 years, (2) considered themselves insufficiently active (ie, not currently achieving the Australian guidelines of 150 min of MVPA/week), (3) were current Facebook users, (4) did not have an existing medical condition for which they had been advised by a doctor to avoid exercise, and (5) were able to speak English.

Interested participants could access the app by typing “Active Team” into the Facebook search function, or by following a link included in the Facebook advertisement. The first page of the app was a welcome page, containing an information video and a detailed participant information sheet. Participants could then use the app to register interest in the study and complete baseline surveys. The app guided participants through the process of inviting eligible Facebook friends to form a team, which resulted in an invitation being posted on relevant friends’ Facebook newsfeeds, along with a link to the app. Participants were formally enrolled into the study if they completed baseline surveys and were part of a team comprising 3 to 8 members. Once a team was finalized, the whole team was randomly allocated to either the intervention or the control condition, using a computer-generated randomization sequence with blocking (block size = six) with allocation concealment. Participants received an automated email informing them of which condition they were enrolled in and when their Active Team challenge would begin.

Teams allocated to the intervention condition received access to the full Active Team app and were mailed a pedometer. Teams allocated to the control condition were placed on a waiting list to receive access to the intervention (app and pedometer) at completion of the study and were told that their health would be monitored over the ensuing 5 months.

### Assessments and Outcome Measures

There were three assessment points for all participants: (1) baseline (at recruitment), (2) 8 weeks (coinciding with the final week of the intervention), and (3) 20 weeks (3-months postcompletion of the intervention). All measurements were completed online. Blinding of participants was not possible, however single blinding was achieved, in that the outcome measures were administered by computer. Thus, the potential for introducing bias was eliminated compared with a scenario where the outcome measures were administered by a person who was aware of the participants’ group allocation.

The primary outcome measure was self-reported total weekly MVPA. This was assessed using the Active Australia Survey (AAS) [25], which records physical activities over the previous 7 days. The validated instrument includes eight items relating to the frequency (four items) and duration (four items) of the following: walking (for exercise, recreation, or transport), vigorous physical activities (such as jogging, cycling, aerobics, and competitive sport), and moderate physical activity (such as gentle swimming, tennis, and golf; excluding walking). As per AAS protocol, total weekly MVPA was calculated as walking time + moderate time + (2 x vigorous time), with each individual item being truncated at a maximum of 840 minutes per week, and total physical activity (PA) being truncated at a maximum of 1680 minutes per week in order to reduce the risk of overreporting [25]. The AAS has been shown to have moderate reliability (rho=.56 to .64) [26] and moderate validity when compared with weekly pedometer step counts (rho = .43) and accelerometry (rho =.52) [26].

Secondary outcomes included examining the physical activity types/intensities separately (ie, weekly walking time, other moderate physical activity, and vigorous physical activity; all derived from the AAS) and quality of life. The impact of the intervention on overall quality of life (and mental health quality of life, in particular), was determined using the Assessment of Quality of Life-6D (AQoL-6D) scale [27], a 20-item instrument assessing six health-related domains. The AQoL-6D has been shown to demonstrate strong test-retest reliability (baseline and 2 weeks, *r*_intraclass correlation (ICC)_ = .88; baseline and 1 month, *r*_ICC_ = .85) [28] and acceptable internal consistency (gamma coefficients [equivalent to standardized correlation coefficients] ranging from .73 to .96 for each subscale; except for sensory perception = .51) [27]. The mental health subscale has good concurrent validity when compared to the 36-item Short Form Health Survey (SF-36) (Pearson’s *r*=.72) [29].

Basic demographic characteristics were also collected: date of birth, sex, highest education level (high school, post-high school [trade/certificate/diploma], university), and self-reported height and weight. The self-reported height and weight information was used to calculate body mass index (BMI), categorized as the following: underweight (<18.5 kg/m^2^), normal (18.5 to <25.0 kg/m^2^), overweight (25.0 to <30.0 kg/m^2^), and obese (≥30.0 kg/m^2^).

Participants’ engagement with the app was assessed via usage statistics, including the number of visits to the app, participants’ step-logging patterns, number of virtual gifts sent, and number of posts on the message walls.

Feasibility of the intervention was determined by using a purpose-designed feedback questionnaire, which was completed by intervention participants during the 8-week assessment. The scale contained nine items, each marked on a 5-point Likert scale—strongly disagree, disagree, neutral, agree, strongly agree. Three items related to perceptions of the overall app (eg, “I think the app is user friendly”), four items related to perceptions of specific features of the app (eg, “I found the daily tips useful”), and two items related to perceptions of the impact of the program (eg, “I felt like my A-Team teammates influenced me to improve my exercise regime”).

### Statistical Analysis

The primary outcome was change in MVPA at 8 weeks. Change in MVPA at 20 weeks and changes in all of the other outcomes at 8 and 20 weeks were considered secondary outcomes. A sample of 106 was required to detect an interaction effect size of Cohen’s d=0.25 (small effect) for the primary outcome, given two groups, three repeated measures, an alpha level of .05, and 80% power (G-Power version 3.1.9.2, Universitat Kiel, Germany, 2014). The sample size was inflated to account for a design effect (potential for clustering of results within teams). Assuming an intracluster correlation coefficient of rho = .01, and approximately 5 participants per team, the design effect was 1+.01(5-1) = 1.04, therefore, the final target was 106 x 1.04 = 110 participants in total.

Participants’ baseline characteristics were analyzed descriptively. Changes in primary and secondary outcomes from baseline to 8 and 20 weeks were analyzed using random-effects mixed modelling. To account for the data structure (participants nested within teams, with three repeated measures), analyses were conducted using Generalized Linear Mixed Models in SPSS version 21, with the individual and the cluster (ie, team) entered as random effects (“unstructured” covariance). The group (ie, intervention vs control), time (ie, baseline, 8 weeks, and 20 weeks), and a group x time interaction term were entered as fixed effects. The intention-to-treat principle was used for data analysis whereby all participants randomized at the commencement of the trial were retained for analysis [30]. Missing data were imputed for the small number of individuals with missing data at posttest (12/110, 10.9% of participants at 8 weeks, and 14/110, 12.7% of participants at 20 weeks) using baseline observations carried forward, which is more conservative and less susceptible to bias than last observation carried forward [31]. Where variables were right skewed (ie, physical activity variables), a log-linear distribution correction was applied.

Usage and feasibility data were analyzed descriptively using frequencies, means, and standard deviations. A small number of predefined subgroup analyses were undertaken to determine whether intervention effectiveness was related to key sociodemographic characteristics (ie, age and sex), baseline physical activity levels, and intervention dosage. Further subgroup analyses were not undertaken to prevent capitalization on chance. Baseline activity levels were categorized as sufficient or insufficient activity, according to the Australian physical activity guideline of ≥ 150 minutes of MVPA per week. Dosage was determined by dichotomizing the number of log-in occasions into low (< 18, 25/51, 49%) and high (≥ 18, 26/51, 51%) based upon a median split. The subgroup analysis was undertaken among the intervention participants only, using Generalized Linear Mixed Models, with total physical activity time entered as the target variable; individual and team entered as random effects; and age, sex, baseline adherence to MVPA guidelines, and intervention dosage entered as fixed effects. Significance for all analyses was set at *P*<.05 without adjustment for multiple comparisons, however exact P values have been reported.

## Results

### Participants

A total of 142 potential participants registered their interest for the study, however, only 110 were in teams that successfully formed, and were formally enrolled into the study. Of the 110 participants, 51 (46.4%) were randomized to the intervention group (12 teams) and 59 (53.6%) to the control group (13 teams). Retention at follow-up was high, with 96 of the 110 (87.3%) participants completing the 20-week follow-up. Out of 110 participants, 4 (3.6%) formally withdrew from the study citing the following reasons: needing to undergo elective surgery for a preexisting condition (intervention group, n=1), lack of time (control group, n=1), overseas vacation (control group, n=1), and too physically active (control group, n=1).

Participants’ demographic and baseline characteristics are shown in Table 1. Of the 110 participants, 78 (70.9%) participants were female, and 46 (41.8%) were within the normal BMI range. There was a relatively even spread of participants across the age group categories. The majority (71/110, 64.5%) of participants were undertaking, or had completed, university education. A total of 59.1% of the sample (65/110) were insufficiently physically active (ie, achieving less than 150 min/week MVPA).

**Table 1:** Descriptive characteristics of the study sample at baseline (n=110).

Baseline characteristics		Intervention (n=51), n (%) or mean (SD)	Control (n=59), n (%) or mean (SD)	Total (n=110), n (%) or mean (SD)
**Age in years, n (%)**				
	18 to <25	12 (24)	14 (24)	26 (23.6)
	25 to <35	17 (33)	15 (25)	32 (29.1)
	35 to <45	12 (24)	17 (29)	29 (26.4)
	45 to 65	10 (20)	9 (15)	19 (17.3)
**Sex, n (%)**				
	Male	14 (27)	12 (20)	26 (23.6)
	Female	37 (73)	45 (76)	82 (74.5)
**BMI** ^a^ **, n (%)**				
	Underweight	2 (4)	1 (2)	3 (2.7)
	Normal	23 (45)	23 (39)	46 (41.8)
	Overweight	12 (24)	18 (31)	30 (27.3)
	Obese	13 (25)	15 (25)	28 (25.5)
**Highest education level, n (%)**				
	High school or lower	5 (10)	11 (19)	16 (14.5)
	Some post-high school (eg, trade or diploma)	11 (22)	10 (17)	21 (19.1)
	University	35 (69)	36 (61)	71 (64.5)
Insufficient PA^b^ (<150 min/week), n (%)		33 (65)	32 (54)	65 (59.1)
Baseline total PA (min/week), mean (SD)		279 (320)	278 (313)	279 (314)
Baseline weekly total AQoL-6D^c^, mean (SD)		0.80 (0.14)	0.82 (0.14)	0.81 (0.14)

^a^BMI: body mass index

^b^Physical activity (PA)—calculated as the sum of weekly walking, moderate, and vigorous physical activity.

^c^Assessment of Quality of Life-6D (AQoL-6D) scale.

### Changes in Physical Activity and Quality Of Life

The results for the primary and secondary outcome measures are shown in s 2 and 3.

Both the intervention and control groups increased their MVPA time from baseline to 8 weeks (primary outcome) (see Table 2). This increase was considerably larger in magnitude for the intervention group relative to the control group—135 minutes of increase relative to the control group (treatment effect size = 0.39, *P*=.03). At 20 weeks, both groups’ physical activity time remained elevated compared with baseline. Relative to the control group, the intervention group appeared to maintain a 41-minute increase (treatment effect size = 0.11), however this was not statistically significant (*P*=.26) (see Table 3).

The secondary physical activity outcomes revealed that the change in overall physical activity at 8 weeks was primarily driven by a change in time spent walking. Relative to the control group, the intervention group increased their walking time by an average of 155 minutes (treatment effect size = 0.69, *P*<.001). There were no significant group x time differences for walking at week 20, and no significant group x time effects for other types of moderate physical activity and vigorous physical activity at either time point.

There were no significant group x time effects for overall quality of life or mental health quality of life at either time point.

**Table 2:** Outcome measures at baseline and at 8-week follow-up.

Outcome measures		Assessment period, mean (SD)		Baseline to 8 weeks		
		Baseline	8 weeks	Mean change (SE)	Treatment effect, effect size (95% CI)	Group-by-time interaction, *F*_1,324_ (*P*)
**Overall PA** ^a^ **time** ^b^ **↑** ^c^						
	Intervention	279 (320)	528 (391)	248 (59)	0.39 (0.01, 0.76)	4.93 (.03)
	Control	278 (313)	391 (371)	113 (43)		
**Walking time ↑**						
	Intervention	127 (198)	332 (289)	205 (38)	0.69 (0.30, 1.07)	13.01 (<.001)
	Control	110 (124)	160 (185)	50 (23)		
**Vigorous PA time ↑**						
	Intervention	52 (102)	78 (138)	26 (20)	0.12 (-0.25, 0.50)	0.89 (.35)
	Control	63 (110)	83 (117)	19 (11)		
**Other moderate PA time ↑**						
	Intervention	50 (127)	73 (154)	23 (29)	0 (-0.37, 0.38)	0.09 (.77)
	Control	46 (128)	68 (171)	22 (23)		
**Overall AQoL-6D** ^d^ **↑**						
	Intervention	0.80 (0.14)	0.81 (0.14)	0.01 (0.01)	0.04 (-0.34, 0.41)	0.26 (.61)
	Control	0.82 (0.14)	0.83 (0.14)	0.01 (0.01)		
**Mental health AQoL-6D ↑**						
	Intervention	0.59 (0.23)	0.62 (0.23)	0.03 (0.03)	0.10 (-0.27, 0.48)	0.02 (.90)
	Control	0.62 (0.25)	0.63 (0.23)	0.01 (0.02)		

^a^PA: physical activity

^b^Time is in minutes/week.

^c^Arrows (↑) indicate the desired direction (increase) of change.

^d^Assessment of Quality of Life-6D (AQoL-6D) scale.

**Table 3:** Outcome measures at baseline and at 20-week follow-up.

Outcome measures		Assessment period, mean (SD)		Baseline to 20 weeks		
		Baseline	20 weeks	Mean change (SE)	Treatment effect, effect size (95% CI)	Group-by-time interaction, *F*_1,324_ (*P*)
**Overall PA** ^a^ **time** ^b^ **↑** ^c^						
	Intervention	279 (320)	376 (377)	97 (50)	0.11 (-0.26, 0.49)	1.29 (.26)
	Control	278 (313)	335 (342)	56 (47)		
**Walking time ↑**						
	Intervention	127 (198)	165 (186)	38 (29)	0.08 (-0.29, 0.46)	1.55 (.21)
	Control	110 (124)	133 (137)	23 (20)		
**Vigorous PA time ↑**						
	Intervention	52 (102)	89 (139)	37 (16)	0.15 (-0.23, 0.52)	1.41 (.24)
	Control	63 (110)	82 (138)	18 (18)		
**Other moderate PA time ↑**						
	Intervention	50 (127)	38 (100)	-12 (21)	-0.07 (-0.44, 0.31)	0.01 (.94)
	Control	46 (128)	43 (95)	-3 (16)		
**Overall AQoL** ^d^ **↑**						
	Intervention	0.80 (0.14)	0.83 (0.15)	0.03 (0.01)	0.36 (-0.02, 0.73)	0.15 (.70)
	Control	0.82 (0.14)	0.82 (0.15)	0.00 (0.01)		
**Mental health AQoL ↑**						
	Intervention	0.59 (0.23)	0.64 (0.23)	0.05 (0.02)	0.36 (-0.02, 0.73)	0.44 (.51)
	Control	0.62 (0.25)	0.61 (0.26)	-0.02 (0.02)		

^a^PA: physical activity

^b^Time is in minutes/week.

^c^Arrows (↑) indicate the desired direction (increase) of change.

^d^Assessment of Quality of Life-6D (AQoL-6D) scale.

### Usage

Of the 51 participants in the intervention group, 48 (94%) used the app at least once. Usage rates were reasonably high; 28 (55%) logged steps for all 50 days of the program as intended, while 35 (69%) logged steps for 36 days or more. These steps were logged across a mean of 18 unique log-in occasions (SD 13.3, range 0-46). On average, intervention participants logged 8867 (SD 2850) steps per day, and one-third of participants (16/51, 31%) met or exceeded the intervention target of 500,000 steps in 50 days. Participants sent a mean of 4.8 gifts (SD 6.3, range 0-27) to their teammates, and made a mean of 2.7 wall posts to their team discussion wall (SD 3.4, range 0-13).

### Feasibility

A total of 47 of the 51 (92%) original intervention participants completed the participant feedback questionnaire at 8 weeks. Feedback about the app overall was generally positive: 32 out of the 47 respondents (68%) either agreed or strongly agreed that the app was user friendly, 32 (68%) liked the overall presentation of the app, and 35 (74%) reported they were able to navigate easily around the app.

Feedback was also sought on the specific features of the app: 38 respondents out of 47 (81%) reported that they found the “My steps” page useful (where participants logged their daily step counts); however, there was less agreement that the daily tips were useful (18/47, 38% agreed/strongly agreed), that the virtual gifts were motivating (14/47, 30% agreed/strongly agreed), or that the unlockable awards were motivating (12/47, 26% agreed/strongly agreed).

Approximately half of the 47 respondents reported that they felt their teammates influenced them to improve their exercise regimen (27/47, 57%) and that the app provided them with social support (21/47, 45%).

### Subgroup Analysis

Subgroup analyses were undertaken to determine whether, within the intervention group, change in MVPA was related to age or sex, intervention “dosage” (high vs low), and achievement of physical activity guidelines at baseline. Results showed that participants’ success in the program was unrelated to sex (*F*_1,41_= 0.10, *P*=.91) and age (*F*_1,41_= 1.17, *P*=.32), however, it was associated with intervention dosage, with “high dose” participants increasing their MVPA significantly more than “low dose” participants (*F*_1,41_ = 3.06, *P*=.04). Furthermore, participants who were insufficiently active at baseline were more likely to increase their MVPA using the program (*F*_1,41_= 466.71, *P*<.001). Of the 33 intervention participants who were insufficiently active at baseline, 21 (64%) were sufficiently active at 8 weeks, and 13 (39%) continued to be sufficiently active at the 20-week follow-up.

No adverse events were reported throughout the trial period.

## Discussion

### Principal Findings

This study found that a 50-day team-based online social networking physical activity intervention incorporating pedometers produced a large and significant change in MVPA (the study’s primary outcome) during the course of the intervention. The change was primarily driven by an increase in time that intervention participants spent walking (155 min/week relative to the control group). However, the intervention participants’ improvements over those of the control participants were not maintained 3 months after the stimulus was removed. There was a pattern for the intervention to favorably impact on overall quality of life and mental health quality of life at the 20-week follow-up, however, this was not statistically significant. The intervention achieved reasonably high rates of engagement and retention, and participant feedback was generally positive.

To our knowledge, this is the first randomized controlled trial evaluating a physical activity intervention delivered via Facebook to report a significant improvement. Two other studies, both utilizing Facebook groups [16,32] to deliver and/or complement a physical activity intervention, reported significant time effects, but not group-by-time effects (ie, in those studies, both the intervention and control groups improved across the course of the intervention, but the degree of improvement did not significantly differ between intervention and control groups). The difference in findings may be due to the way in which Facebook was used between these two studies and our current study. Cavallo and colleagues [16] and Valle and colleagues [32] set up private Facebook groups that were intended to facilitate discussion and sharing of information between intervention participants. In contrast, our intervention involved a Facebook app (ie, standalone software, delivered via the Facebook platform) that focused on assisting participants to log, track, and compare their daily physical activity with other users. Furthermore, in both the Cavallo and colleagues [16] and Valle and colleagues [32] studies, participants who were strangers to each other offline were intended to communicate with each other via the online groups. In contrast, our study drew upon existing online social networks, so that study participants were interacting within teams of people with whom they shared an existing online connection, and presumably an offline connection as well. This is arguably more consistent with the use of online social networks, given that people typically use Facebook to interact with people with whom they share an offline connection [33]. The approach used in our study was somewhat similar to that reported by Foster and colleagues [15], whereby a group of workmates compete in a pedometer-based challenge. Similar to our study, they reported physical activity behavior change of a large magnitude.

As with many Web-based physical activity interventions [4], recidivism was apparent in the current study. At the end of the program, there was a more than 2-hour difference in weekly physical activity between groups, yet 12 weeks later at the 20-week follow-up assessments, the intervention participants’ physical activity levels had returned to within 40 minutes of those of the control group (note that the study was insufficiently powered for a 40-minute difference to be statistically significant). Despite the lack of statistical significance, the trend for change provides insight for a future, larger study, as a change of this magnitude is likely to be of clinical significance. Furthermore, the subgroup analysis, which showed that around 40% of participants who were insufficiently active at baseline successfully met physical activity guidelines at the 20-week follow-up, suggests that the population impact of the app may be considerable if the intervention was to be implemented at a large scale.

Further work is required to determine how to maintain physical activity behavior change achieved by the Active Team intervention in the longer term. Fjeldsoe and colleagues’ [34] review of physical activity behavior change maintenance suggests that increasing the intervention’s duration, and/or building long-term follow-up prompts into the app may be useful in achieving this. While the study’s intervention was mainly guided by the theory of planned behavior, other behavior change theories which emphasize behavior maintenance, such as the transtheoretical model [35] or the Health Action Process Approach [36], and self-regulation theories [37] may provide valuable insights into further strategies to maintain behavior change in the longer term.

Gamification has been a popular tech trend in recent years. The Active Team app was carefully designed to incorporate numerous gamification features; however, usage statistics and participant feedback specific to these features suggested they were not strongly embraced by participants. Despite this, the app overall achieved strong usage and participant feedback. It is possible that the influence of gamification was larger than participants indicated—that it worked in a subconscious way and did, in fact, contribute to engagement and utility of the app. Alternatively, it may be that gamification has been overhyped, or at least unsuccessful in the form in which it was implemented in our app. Such hypotheses cannot be answered by our study; indeed, the field of gamification for health behavior change is in its infancy and considerable further work is needed to explore its efficacy and optimal application.

Facebook is recognized to have extremely diverse reach, appealing to users of widely varying sociodemographic backgrounds [38,39]. Nevertheless, our study recruited a predominantly female, middle-class sample. Further work is required to determine how to attract a diverse sample, and in particular, increase reach to low physical activity/low socioeconomic status users, who are likely to gain the most benefit from a physical activity intervention. Furthermore, once effective intervention approaches have been devised, research focused on determining how to disseminate interventions on a mass scale will be key. Insights offered by social marketers and traditional marketers are likely to be highly valuable in achieving these goals.

### Strengths

Strengths of the current study are the novelty of the intervention, which used online social networking to recruit participants and deliver a physical activity program (in combination with a pedometer), that the app incorporated novel features (gamification and fun), and that there was minimal contact from research personnel. The online intervention itself was delivered entirely via the software and automated emails. This hands-off delivery approach can facilitate large-scale dissemination of the intervention in the future. Further strengths of the study were the randomized controlled trial study design, and the relatively high rates of compliance and retention achieved.

### Limitations

For logistical reasons, the study used self-reported measures of physical activity, and these are typically considered to be susceptible to social desirability bias [40]. Interestingly, Crutzen and Goritz [41] recently examined this issue in over 5000 participants, and found social desirability bias was, in fact, unrelated to Web-based self-reported physical activity, suggesting that Web-based self-reports of physical activity are more trustworthy and useful. An advantage of self-reported physical activity, as opposed to objectively measured physical activity, is the considerably lower participant assessment burden, which arguably enhances the study’s ecological validity. Similarly, in the interest of minimizing assessment burden, we did not measure theory of planned behavior constructs and, hence, were unable to determine whether changes in these constructs explain intervention effects. In the future, a more extensive measurement protocol, including such measures, would provide useful insights into possible mechanisms. We decided not to exclude participants who stated that they obtained less than 150 minutes of weekly MVPA at enrolment, yet who went on report more than 150 minutes during the baseline surveys. In order to allow the intervention’s social and team nature to function as intended, it was important to allow participants to undertake the intervention with friends, without applying too many restrictions. The application of RCT principles, such as strict eligibility criteria and prevention of contamination, in online social network interventions presents researchers with many dilemmas, and we would argue that a degree of pragmatism is required to allow the social networking intervention to function as intended, and consequently produce results that are useful in the “real world.” As with most health behavior randomized controlled trials, blinding of participants to the intervention arm was not possible, however blinding of assessors was achieved since all assessments were delivered via online surveys. Additionally, this intervention had two components—the use of (1) a pedometer and (2) the app—and the use of a wait-list control meant that the individual influence of these components on study outcomes cannot be determined in the current study. Kang and colleagues’ [42] meta-analysis of pedometer-based interventions found an overall effect size of 0.68 for daily step count—very similar to the effect size of 0.69 found for walking time in our study. Thus, it is possible that the pedometer component largely accounted for the behavior change observed in our study. However, engagement data indicated high use of the app, suggesting the combined intervention elements (ie, pedometer and app) were both important in achieving behavioral change. Finally, the subgroup analyses were likely underpowered, and the sample may not necessarily be generalizable given the high proportion of female and well-educated individuals.

The “snowball”-style recruitment method offers both strengths and weaknesses. Firstly, it is in keeping with how information and communication typically spread via online networks. Furthermore, it may have alleviated the problem encountered by many physical activity interventions in that they tend to attract relatively motivated individuals. It is likely that the team captains themselves may have been the "typical" motivated individuals who volunteer for research projects, however, it is plausible that the team members would not otherwise have joined, except that they received an invitation from their friend—this use of social influence is often termed “word-of-mouth seeding” in marketing [43]. An unanticipated drawback on the team structure was that numerous potential participants who registered could not be formally enrolled because their team never formed. Future iterations of the Active Team software will explore alternative recruitment structures in order to draw on the positives of snowball recruitment, without the present limitations of the strict team structure.

### Conclusions

This study has provided preliminary evidence that an online, social networking physical activity intervention using pedometers can produce sizeable short-term physical activity change. Future work is needed to determine how to maintain behavior change in the longer term, how to reach underserved populations on this platform, and how to disseminate such interventions on a mass scale.

## Appendix

Multimedia Appendix 1 CONSORT-EHEALTH checklist V1.6.2 [44].

## Abbreviations

AAS: Active Australia Survey
AQoL-6D: Assessment of Quality of Life-6D
BMI: body mass index
ICC: intraclass correlation
MVPA: moderate-to-vigorous physical activity
PA: physical activity
RCT: randomized controlled trial
SF-36: 36-item Short Form Health Survey
