# Supplementary material for: A Web-Based, Social Networking Physical Activity Intervention for Insufficiently Active Adults Delivered via Facebook App: Randomized Controlled Trial
Source: J Med Internet Res. 2015 Jul 13;17(7):e174. doi: 10.2196/jmir.4086 (PMC4526990; doi:10.2196/jmir.4086)
Supplement: Multimedia Appendix 1 [file jmir_v17i7e174_app1.pdf]

CONSORT-EHEALTH Checklist V1.6.2 Report

(based on CONSORT-EHEALTH V1.6), available at [http://tinyurl.com/consort-ehealth-v1-6].

4086

Date completed

11/24/2014 20:12:13

by

Monika Ferguson

A web-based social networking physical activity intervention for inactive adults delivered via a Facebook application: a randomized controlled trial.

TITLE

1a-i) Identify the mode of delivery in the title

A web-based social networking physical activity intervention for inactive adults delivered via a Facebook application: a randomized controlled trial.

1a-ii) Non-web-based components or important co-interventions in title

N/A - the study does not include a non-web-based component.

1a-iii) Primary condition or target group in the title

"A web-based social networking physical activity intervention for inactive adults delivered via a Facebook application: a randomized controlled trial."

ABSTRACT

1b-i) Key features/functionalities/components of the intervention and comparator in the METHODS section of the ABSTRACT

“Teams were randomly allocated to receive access to a 50-day online social networking physical activity intervention which included self-monitoring and social elements (“Active Team” Facebook application; n=51 individuals, n=12 teams) or a wait-listed control condition (n=59 individuals, n=13 teams)."

1b-ii) Level of human involvement in the METHODS section of the ABSTRACT

“110 adults (mean age 35.6, SD 12.4 y) were recruited online in teams of 3-8 friends. Teams were randomly allocated to receive access to a 50-day online social networking physical activity intervention which included self-monitoring and social elements (“Active Team” Facebook application; n=51 individuals, n=12 teams) or a wait-listed control condition (n=59 individuals, n=13 teams). Assessments were undertaken online at baseline, 8 weeks and 20 weeks.”

1b-iii) Open vs. closed, web-based (self-assessment) vs. face-to-face assessments in the METHODS section of the ABSTRACT

“110 adults (mean age 35.6, SD 12.4 y) were recruited online in teams of 3-8 friends...Assessments were undertaken online at baseline, 8 weeks and 20 weeks.”

1b-iv) RESULTS section in abstract must contain use data

Mentioned in methods section of abstract: “110 adults (mean age 35.6, SD 12.4 y) were recruited online in teams of 3-8 friends. Teams were randomly allocated to receive access to a 50-day online social networking physical activity intervention which included self-monitoring and social elements (“Active Team” Facebook application; n=51 individuals, n=12 teams) or a wait-listed control condition (n=59 individuals, n=13 teams).”

1b-v) CONCLUSIONS/DISCUSSION in abstract for negative trials

N/A – primary outcome changed in short term: “At 8 week follow up, the intervention participants had significantly increased their total weekly MVPA by 135 minutes relative to the control group (P = 0.03), due primarily to increases in walking time (155 min/week increase relative to controls; P < .001).”

INTRODUCTION

2a-i) Problem and the type of system/solution

The introduction explains that physical activity is a major public health concern and that population-level interventions, such as web-based physical activity interventions, are required to address this:

"Physical inactivity is a leading modifiable cause of death and disease worldwide and causes as many deaths as smoking [1]. Just 30 minutes a day of moderate intensity physical activity halves the risk of leading causes of morbidity and mortality, such as cardiovascular disease, type 2 diabetes and obesity, and reduces the risk of breast and bowel cancer, depression and anxiety [2]. Despite this, many people in developed countries are insufficiently active to achieve these benefits. For example, in Australia, an estimated 67% of adults get less than 30 minutes physical activity a day [3]. Population-based interventions are needed to assist the general adult population to become more physically active.

Web-based physical activity interventions offer an opportunity to reach a large number of people at relatively low cost. Systematic reviews and meta-analyses of web-based physical activity interventions demonstrate they are effective in changing behavior [4, 5], however, typically have not been adopted by large numbers of users and appear to have difficulty sustaining user engagement over an extended period [4, 5]. New intervention approaches capitalizing on recent technology trends, such as online social networks and gamification, may assist in overcoming these issues."

2a-ii) Scientific background, rationale: What is known about the (type of) system

"Online social networks reportedly account for one-quarter of all time spent online [6, 7], and appear to offer considerable potential for delivery of public health campaigns, for several reasons. First, they can reach very large audiences (e.g., Facebook, the world’s largest social networking website, had 1.32 billion unique users each month as of June 2014) [8]. Second, messages can be delivered via existing contacts, which may be more influential than health messages delivered via traditional marketing strategies [9]. Third, unlike traditional web-based interventions [4], online social networks typically achieve high levels of user engagement and retention [10].

Another online trend that has emerged in recent years is “gamification”. Gamification refers to the application of videogame elements, such as fun, challenges, competition and rewards, in non-gaming situations [11]. In the commercial sector, such techniques have reported to markedly increase engagement (e.g., a software company reported an 8-fold increase in user engagement after introduction of gamification features) [12]. A recent systematic review of health behavior change interventions delivered using online social networks [13] found that compared to studies which did not incorporate gamification features, the one study that did (in the form of competition between users [14]) achieved substantially larger intervention effects and higher levels of user engagement.

To date, only a handful of studies have attempted to use existing popular online social network platforms (such as Facebook and Twitter) to intervene on physical activity. The most common approach has been the use of a Twitter feed or private Facebook groups to share content regarding physical activity and facilitate discussion between study participants [13]. In most cases, the online social network intervention has been provided as a component, complementing a more comprehensive intervention package, for example, involving access to a physical activity self-monitoring website with personalized feedback from a health professional [15]; provision of pedometer, digital scales, cooking equipment and personalized feedback [16]; or access to a series of podcasts, advice from an expert moderator, and a calorie counting application (app) [17]. To our knowledge, only one previous study [14] has utilized a Facebook app (i.e., software created by third party developers to function within the Facebook platform and access data in Facebook) to intervene on physical activity."

METHODS

3a) CONSORT: Description of trial design (such as parallel, factorial) including allocation ratio

“The primary objective of the study was to determine whether a team-based 50-day social networking physical activity intervention delivered via a Facebook app and incorporating gamification features was effective in changing weekly moderate to vigorous physical activity (MVPA) in adults aged 18-65 y. The secondary objectives were 1) to determine whether the intervention impacted other physical activity (weekly walking, vigorous physical activity and moderate physical activity time), and quality of life (in particular, mental quality of life), 2) to determine usage, and 3) to examine the feasibility of the online intervention.”

3b) CONSORT: Important changes to methods after trial commencement (such as eligibility criteria), with reasons

N/A – no changes were made to methods after trial commencement.

3b-i) Bug fixes, Downtimes, Content Changes

N/A – no bug fixes, downtimes or content changes after trial commencement.

4a) CONSORT: Eligibility criteria for participants

"Participants were eligible if they met the following criteria: (1) were aged between 18 and 65 years; (2) considered themselves insufficiently active (i.e., not currently achieving the Australian guidelines of 150 min of MVPA/week); (3) were current Facebook users; (4) did not have an existing medical condition for which they had been advised by a doctor to avoid exercise; and (5) were able to speak English."

4a-i) Computer / Internet literacy

Computer/internet literacy was not explicitly states as it was encompassed by the inclusion criterion of participants being current Facebook users (which requires an understanding of how to use a computer and the Internet).

4a-ii) Open vs. closed, web-based vs. face-to-face assessments:

"Participants were recruited through a Facebook advertising campaign, media stories in the local newspaper and television news bulletin, and distribution of flyers at the University of South Australia campuses…Interested participants could access the app by typing "Active Team" into the Facebook search function, or by following a link included in the Facebook advertisement. The first page of the app was a welcome page, containing an information video and a detailed participant information sheet. Participants could then use the app to register interest in the study and complete baseline surveys. The app guided participants through the process of inviting eligible Facebook friends to form a team, which resulted in an invitation being posted on relevant friends' Facebook newsfeeds, along with a link to the app. Participants were formally enrolled into the study if they completed baseline surveys and were part of a team comprising 3-8 members."

4a-iii) Information giving during recruitment

"Participants provided informed consent online prior to commencing the study…Interested participants could access the app by typing "Active Team" into the Facebook search function, or by following a link included in the Facebook advertisement. The first page of the app was a welcome page, containing an information video and a detailed participant information sheet."

4b) CONSORT: Settings and locations where the data were collected

N/A – all data were collected online

4b-i) Report if outcomes were (self-)assessed through online questionnaires

"All measurements were completed online."

4b-ii) Report how institutional affiliations are displayed

Institutional affiliation (University of South Australia) was displayed on the recruitment material (flyers and Facebook advertising), Active Team welcome video, and the participant information sheet.

5) CONSORT: Describe the interventions for each group with sufficient details to allow replication, including how and when they were actually administered

5-i) Mention names, credential, affiliations of the developers, sponsors, and owners

See Acknowledgements section:
"The authors thank Portal Australia for assisting with the development of the Active Team application..."

5-ii) Describe the history/development process

N/A - this study was designed to be a preliminary investigation of the Active Team app, and findings of this study will be used to guide the development of future versions of the app.

5-iii) Revisions and updating

N/A - the Active Team app and intervention did not undergo any major changes during the trial.

5-iv) Quality assurance methods

N/A - the app was developed in conjunction with Portal Australia, a software development company providing services to a range of healthcare providers in Adelaide, Australia.

5-v) Ensure replicability by publishing the source code, and/or providing screenshots/screen-capture video, and/or providing flowcharts of the algorithms used

Screenshots have been provided in Figures 1 and 2, and other key details of the Active Team app have been provided in the "Intervention" section. Given that this study was designed as a preliminary exploration of the efficacy, engagement, and feasibility of the Active Team app, it is anticipated that the future version of the app and intervention will be updated based on the findings of this study and therefore it is unlikely that we or other researchers would replicate the study with the version of the app in its present form. If researchers do wish to replicate the app, they can contact the first author for additional screen shots or other information.

5-vi) Digital preservation

As per response 5-v, it is likely that the version of the Active Team app used in the present study will be updated. As such, screenshots have been provided in the manuscript (see Figures 1 and 2).

5-vii) Access

""Active Team" is a new, free 50-day team-based Facebook app, developed to assist adults to increase their physical activity levels… Interested participants could access the app by typing "Active Team" into the Facebook search function, or by following a link included in the Facebook advertisement. The first page of the app was a welcome page, containing an information video and a detailed participant information sheet. Participants could then use the app to register interest in the study and complete baseline surveys."

5-viii) Mode of delivery, features/functionalities/components of the intervention and comparator, and the theoretical framework

""Active Team" is a new, free 50-day team-based Facebook app, developed to assist adults to increase their physical activity levels. Participants are provided with a pedometer, and encouraged to achieve 10,000 steps per day [18], working in teams of 3-8 existing Facebook friends. Active Team is designed to encourage friendly rivalry within friendship groups, offer peer encouragement and support, and be quick, social and enjoyable to use. It includes a calendar to log daily step counts (steps can be logged up to 7 days in arrears; Figure 1), a team tally board to allow users to monitor their own and their team mates' progress (Figure 2); a team message board for team members to communicate with one another; daily tips for increasing physical activity; gamification features, such as awards for individual and team step logging and step count achievements; and the ability to send virtual gifts to team mates. Automated computer-tailored weekly emails are sent to participants summarizing their progress and encouraging continued participation. Apart from provision of a pedometer, the Active Team intervention approach was designed to be minimally resource-intensive, and therefore did not include provision of extensive instrumental support, expert moderation or feedback from a health professional.

The content and features of Active Team were informed by the Theory of Planned Behavior [19, 20] and Fun Theory [21]. The Theory of Planned Behavior posits that a person's decision to perform a particular behavior is influenced by three factors: attitude, subjective norms and perceived behavioral control [19, 20]. Fun theory advocates that people will be more motivated to do routine activities if they are adapted to be fun [21]. The Active Team app attempts to address each of these factors by: providing daily tips for physical activity, written by a comedian (Theory of Planned Behavior – attitudes and perceived behavioral control; Fun Theory); use of teams for peer encouragement and support (Theory of Planned Behavior – subjective norms; Fun Theory); and setting small achievable goals (daily step count) which are recorded and contribute to a long term/overall goal (500,000 steps) (Theory of Planned Behavior – attitude and perceived behavioral control), unlockable awards, named by a comedian (Fun Theory), and the ability to send virtual gifts, such as a high five and a pink leotard (Theory of Planned Behavior – subjective norms; Fun Theory)."

5-ix) Describe use parameters

As per response 5-viii, participants were encouraged to use the pedometer and the app to achieve 10,000 steps per day. They were also encouraged to use the app to interact with and support their Facebook friends. No limitations on usage were set (participants were encouraged to use the app frequently, and one of the aims of this study was to document usage of/engagement with the app).

5-x) Clarify the level of human involvement

Active Team was designed to use minimal human resources for recruitment, the intervention, and data collection: "Apart from provision of a pedometer, the Active Team intervention approach was designed to be minimally resource-intensive, and therefore did not include provision of extensive instrumental support, expert moderation or feedback from a health professional." For each week of the intervention, automated computer-tailored emails were sent to participants; these included researcher contact details for any participant concerns.

5-xi) Report any prompts/reminders used

As per response 5-x, weekly emails were sent to participants in the intervention group (for the 8 weeks of the program). The purpose of these emails were to encourage participants to continue to be physically active, and to use the app to log their steps and interact with their teammates.

5-xii) Describe any co-interventions (incl. training/support)

N/A – there were no co-interventions.

6a) CONSORT: Completely defined pre-specified primary and secondary outcome measures, including how and when they were assessed

"There were three assessment points for all participants: (1) baseline (at recruitment); (2) 8 weeks (coinciding with the final week of the intervention); and (3) 20 weeks (3 months post completion of the intervention). All measurements were completed online.

The primary outcome measure was self-reported total weekly MVPA. This was assessed using the Active Australia Survey (AAS)[22] which records physical activities over the previous seven days. The validated instrument includes eight items relating to the frequency (four items) and duration (four items) of: walking (for exercise, recreation or transport); vigorous physical activities (such as jogging, cycling, aerobics and competitive sport); and moderate physical activity (such as gentle swimming, tennis and golf; excluding walking). As per AAS protocol, total weekly MVPA was calculated as walking time + moderate time + (2 x vigorous time), with each individual item being truncated at a maximum of 840 minutes per week, and total PA being truncated at a maximum of 1680 minutes per week, in order to reduce the risk of over-reporting.[22] The AAS has been shown to have moderate reliability (r= 0.56 to 0.64)[23] and moderate validity when compared with weekly pedometer step counts (rho = 0.43) and accelerometry (rho = 0.52).[23]

Secondary outcomes included examining the physical activity types/intensities separately (i.e., weekly walking time, other moderate physical activity, and vigorous physical activity; all derived from the AAS), and quality of life. The impact of the intervention on overall quality of life (and mental health quality of life in particular), was determined using the Assessment of Quality of Life – 6D scale (AQoL-6D),[24] a 20-item instrument, assessing six health-related domains. The AQoL-6D has been shown to demonstrate strong test-retest reliability (baseline and 2 weeks, ricc = .88; baseline and 1 month, ricc = .85)[25] and acceptable internal consistency (gamma coefficients [equivalent to standardized correlation coefficients] ranging from .73 – .96 for each sub-scale; except for sensory perception = .51).[24] The mental health sub-scale has good concurrent validity when compared to the 36-item Short Form Health Survey (SF-36; Pearson's r = .72).[26]"

6a-i) Online questionnaires: describe if they were validated for online use and apply CHERRIES items to describe how the questionnaires were designed/deployed

As per response 6a, previously validated, self-report outcome measures were used (although these have not been specifically validated for use in online, application-based health interventions, given the very recent emergence of these interventions in research).

6a-ii) Describe whether and how "use" (including intensity of use/dosage) was defined/measured/monitored

As per the aims of the study, usage of the Active Team app was investigated. This was explored "via usage statistics, including the number of visits to the app, participants' step logging patterns, number of virtual gifts sent, and number of posts on the message walls." Further, "A small number of pre-defined sub-group analyses were undertaken to determine whether intervention effectiveness was related to…intervention dosage…Dosage was determined by dichotomising number of log-in occasions into low (< 18) and high (≥ 18)."

6a-iii) Describe whether, how, and when qualitative feedback from participants was obtained

As per the aims of the study, feedback from participants was sought "by using a purpose-designed feedback questionnaire (nine rating scale items) completed by intervention participants during the 8 week assessment. Three items related to perceptions of the overall app (e.g., "I think the app is user-friendly"), four items related to perceptions of specific features of the app (e.g., "I found the daily tips useful"), and two items related to perceptions of the impact of the program (e.g., "I felt like my A-Team teammates influenced me to improve my exercise regime")."

6b) CONSORT: Any changes to trial outcomes after the trial commenced, with reasons

N/A – there were no changes to the trial outcomes after the trial commenced.

7a) CONSORT: How sample size was determined

7a-i) Describe whether and how expected attrition was taken into account when calculating the sample size

"A sample of 106 was required to detect an interaction effect size of Cohen's d=.25 (small effect) for the primary outcome (MVPA), given two groups, three repeated measures, an alpha level of .05 and 80% power (G-Power Version 3.1.9.2, Universitat Kiel, Germany, 2014). The sample size was inflated to account for a design effect (potential for clustering of results within teams). Assuming an intra-cluster correlation coefficient of ρ = 0.01, and approximately 5 participants per team, the design effect was 1+0.01(5-1) = 1.04, therefore the final target was 106 x 1.04 = 110 participants in total."

7b) CONSORT: When applicable, explanation of any interim analyses and stopping guidelines

N/A – there were no interim analyses.

8a) CONSORT: Method used to generate the random allocation sequence

"Once a team was finalized, the whole team was randomly allocated to either the intervention or the control condition, using a computer-generated randomization sequence with blocking (block size = six)."

8b) CONSORT: Type of randomisation; details of any restriction (such as blocking and block size)

As per response 8a, blocking was used (block size = six) during the randomization process.

9) CONSORT: Mechanism used to implement the random allocation sequence (such as sequentially numbered containers), describing any steps taken to conceal the sequence until interventions were assigned

As per response 8a, random allocation was achieved via a "computer-generated randomization sequence with blocking (block size = six)."

10) CONSORT: Who generated the random allocation sequence, who enrolled participants, and who assigned participants to interventions

"Once a team was finalized, the whole team was randomly allocated to either the intervention or the control condition, using a computer-generated randomization sequence with blocking (block size = six). Participants received an automated email informing them of which condition they were enrolled in and when their Active Team challenge would begin."

11a) CONSORT: Blinding - If done, who was blinded after assignment to interventions (for example, participants, care providers, those assessing outcomes) and how

11a-i) Specify who was blinded, and who wasn't

As stated in the Discussion, "blinding of participants to the intervention arm was not possible, however blinding of assessors was achieved since all assessments were delivered via online surveys."

11a-ii) Discuss e.g., whether participants knew which intervention was the "intervention of interest" and which one was the "comparator"

The participant information sheet specified that the purpose of the study was to test the effectiveness of the Active Team app, and that participants allocated to the intervention group would receive access to the Active Team app immediately and that the control group would receive access at the end of the study.

11b) CONSORT: If relevant, description of the similarity of interventions

N/A – not relevant for ehealth trials.

12a) CONSORT: Statistical methods used to compare groups for primary and secondary outcomes

“Changes in primary and secondary outcomes from baseline to 8 and 20 weeks were analyzed using random-effects mixed-modelling. To account for the study design (participants nested within teams), analyses were conducted using Generalised Linear Mixed Models in SPSS Version 21, with the individual and the cluster (i.e., team) entered as random effects, and the group (i.e., intervention vs control), time and a group x time interaction term entered as fixed effects. The intention to treat principle was used for data analysis whereby all participants randomized at the commencement of the trial were retained for analysis, [27] with baseline scores fed-forward for the small number of individuals with missing data at post-test (n=12 (11%) participants at 8 weeks, and n=14 (13%) participants at 20 weeks). Where variables were right skewed (physical activity variables) a log-linear distribution correction was applied.”

12a-i) Imputation techniques to deal with attrition / missing values

N/A - No imputation techniques were applied, however the intention to treat principle was used.

12b) CONSORT: Methods for additional analyses, such as subgroup analyses and adjusted analyses

“A small number of pre-defined sub-group analyses were undertaken to determine whether intervention effectiveness was related to key socio-demographic characteristics (age and sex), baseline physical activity levels and intervention dosage. Further sub-group analyses were not undertaken to prevent capitalisation on chance. Baseline activity levels were categorised as sufficiently or insufficiently activity (according to the Australian physical activity guideline of ≥ 150 minutes of MVPA per week). Dosage was determined by dichotomising number of log-in occasions into low (< 18) and high (≥ 18).The sub-group analysis was undertaken amongst the intervention participants only, using Generalised Linear Mixed Models, with total physical activity time entered as the target variable, individual and team entered as random effects, and age, sex, baseline adherence to MVPA guidelines and intervention dosage entered as fixed effects.”

RESULTS

13a) CONSORT: For each group, the numbers of participants who were randomly assigned, received intended treatment, and were analysed for the primary outcome

See CONSORT flowchart (Figure 3)

13b) CONSORT: For each group, losses and exclusions after randomisation, together with reasons

“A total of 142 potential participants registered their interest for the study, and 110 were successfully formed into teams and formally enrolled into the study. Of the 110 participants, 51 were randomized to the intervention group (n=12 teams) and 59 to the control group (n=13 teams). Of these, 98 (89.1%) completed the 8 week assessments, and 96 (87.3%) completed the 20 week follow-up. Four participants formally withdrew from the study citing the following reasons: needing to undergo elective surgery for pre-existing condition (intervention group; n=1), lack of time (control group; n=1), overseas vacation (control group; n=1) and too physically active (control group; n=1).”

13b-i) Attrition diagram

See Figure 3.

14a) CONSORT: Dates defining the periods of recruitment and follow-up

“Data collection took place between September 2013 and July 2014.”

14a-i) Indicate if critical “secular events” fell into the study period

N/A – no critical events.

14b) CONSORT: Why the trial ended or was stopped (early)

N/A – the trial was not stopped early.

15) CONSORT: A table showing baseline demographic and clinical characteristics for each group

See Table 1.

15-i) Report demographics associated with digital divide issues

Age, gender and education level are reported in Table 1.

16a) CONSORT: For each group, number of participants (denominator) included in each analysis and whether the analysis was by original assigned groups

16-i) Report multiple “denominators” and provide definitions

The number of participants is specified throughout the Method and Results sections (e.g. Figure 3 and Table 1).

16-ii) Primary analysis should be intent-to-treat

In the Methods section, it is stated that the intention-to-treat method was used for the primary analysis, and that sub-group analysis was conducted with intervention group participants only.

17a) CONSORT: For each primary and secondary outcome, results for each group, and the estimated effect size and its precision (such as 95% confidence interval)

See Table 2 and “Changes in physical activity and quality of life” section:

"Both the intervention and control groups increased their MVPA time (primary outcome) from baseline to 8 weeks. This increase was considerably larger in magnitude for the intervention group relative to the control group (135 minutes of relative increase over the control group, treatment effect size = 0.39), and mixed modelling group x time analyses revealed that the difference was statistically significant (P = .03). At 20 weeks, both groups’ physical activity time remained elevated compared with baseline. Relative to the control group, the intervention group appeared to maintain a 41 minute increase (treatment effect size = 0.11), however this was not statistically significant (group x time effect P = .26).

The secondary physical activity outcomes revealed that the change in overall physical activity at 8 weeks was primarily driven by a change in time spent walking. Relative to the control group, the intervention group increased their walking time by an average of 155 minutes (treatment effect size = 0.69; group x time effect P < .001). There were no significant group x time differences for walking at week 20, and no significant group x time effects for other types of moderate physical activity and vigorous physical activity at either time point."

17a-i) Presentation of process outcomes such as metrics of use and intensity of use

"Of the 51 participants in the intervention group, 48 (94%) used the app at least once. Usage rates were reasonably high; 28 (55%) logged steps for all 50 days of the program as intended, while 35 (70%) logged steps for 36 days or more. These steps were logged across a mean of 18 unique login occasions (SD 13.3, range 0-46). On average, intervention participants logged 8867 (SD 2850) steps per day, and one third of participants (n = 16) met or exceeded the intervention target of 500,000 steps in 50 days. Participants sent a mean of 4.8 gifts (SD 6.3, range 0 – 27) to their team mates, and made a mean of 2.7 wall posts to their team discussion wall (SD 3.4, range 0 – 13)."

17b) CONSORT: For binary outcomes, presentation of both absolute and relative effect sizes is recommended

N/A - primary and secondary outcomes were continuous.

18) CONSORT: Results of any other analyses performed, including subgroup analyses and adjusted analyses, distinguishing pre-specified from exploratory

"Subgroup analyses were undertaken to determine whether, within the intervention group, change in MVPA was related to age or sex, intervention “dosage” (high vs low) and achievement of physical activity guidelines at baseline. Results showed that participants’ success in the program was unrelated to sex (P = .86) and age (P = .50), however it was associated with intervention dosage, with “high dose” participants increasing their MVPA significantly (P = .04) more than “low dose” participants. Furthermore, participants who were insufficiently active at baseline were more likely to increase their MVPA using the program (P < .001). Of the 33 intervention participants who were insufficiently active at baseline, 21 (or 64%) were sufficiently active at 8 weeks, and 13 (40%) continued to be sufficiently active at 20 week follow up."

18-i) Subgroup analysis of comparing only users

As per response 18, subgroup analyses were undertaken with intervention group participants only.

19) CONSORT: All important harms or unintended effects in each group

N/A – “No adverse events were reported throughout the trial period.”

19-i) Include privacy breaches, technical problems

N/A – no privacy breaches or technical problems were experienced.

19-ii) Include qualitative feedback from participants or observations from staff/researchers

“A total of 47 of the 51 original intervention participants completed the participant feedback questionnaire at 8 weeks. Feedback about the app overall was generally positive: 32 out of the 47 respondents (68%) either agreed or strongly agreed that the app was user-friendly, 32 (68%) liked the overall presentation of the app, and 35 (75%) reported they were able to navigate easily around the app.

Feedback was also sought on the specific features of the app: 38 respondents (81%) reported that they found the “My steps” page useful (where participants logged their daily step counts); however there was less agreement that the daily tips were useful (n=18; 38% agreed/strongly agreed), that the virtual gifts were motivating (n=14; 30% agreed/strongly agreed) or that the unlockable awards were motivating (n=12; 26% agreed/strongly agreed).

Approximately half of the 47 respondents reported that they felt their teammates influenced them to improve their exercise regime (n=27; 57%) and that the app provided them with social support (n=17; 45%).”

DISCUSSION

20) CONSORT: Trial limitations, addressing sources of potential bias, imprecision, multiplicity of analyses

20-i) Typical limitations in ehealth trials

“Limitations of the study should also be acknowledged. For logistical reasons, the study used self-reported measures of physical activity, and these are typically considered to be susceptible to social desirability bias [35]. Interestingly, Crutzen and Goritz [36] recently examined this issue in over 5,000 participants, and found social desirability bias was, in fact, unrelated to web-based self-reported physical activity, suggesting that web-based self-reports of physical activity are more trustworthy and useful. An advantage of self-report physical activity, as opposed to objectively measured physical activity, is the considerably lower participant assessment burden, which arguably enhances the study’s ecological validity. As with most health behavior randomized controlled trials, blinding of participants to the intervention arm was not possible, however blinding of assessors was achieved since all assessments were delivered via online surveys. Additionally, this intervention had two components – the use of a pedometer and the app – yet the individual influence of these on study outcomes was not explored. Engagement data indicate high use of both features (with over half of participants using the app to log steps for all days and, on average, logging steps on 18 unique login occasions over the 50 day period), suggesting the combined importance of these elements. Finally, the sample may not necessarily be generalizable given the high proportion of female and well-educated individuals.”

21) CONSORT: Generalisability (external validity, applicability) of the trial findings

21-i) Generalizability to other populations

“…the sample may not necessarily be generalizable given the high proportion of female and well-educated individuals.”

21-ii) Discuss if there were elements in the RCT that would be different in a routine application setting

This was an online intervention designed to be delivered with minimal direct researcher involvement; as such, the RCT would have more human involvement if it were conducted as an in-person intervention. This was highlighted in the Discussion:
"Strengths of the current study are the novelty of the intervention, which used online social networking to recruit participants and deliver the physical activity program, that the app incorporated novel features (gamification and fun), and the minimal contact from research personnel. The intervention itself was delivered entirely via the software and automated emails. This hands-off delivery approach can facilitate large scale dissemination of the intervention in the future."

22) CONSORT: Interpretation consistent with results, balancing benefits and harms, and considering other relevant evidence

22-i) Restate study questions and summarize the answers suggested by the data, starting with primary outcomes and process outcomes (use)

“This study found that a 50-day team-based online social networking physical activity intervention produced a large and significant change in MVPA (the study’s primary outcome) during the course of the intervention. The change was primarily driven by an increase in time intervention participants spent walking (155 min/week relative to the control group). However, the intervention participants’ improvements over the control participants’ were not maintained 3 months after the stimulus was removed. There was a pattern for the intervention to favorably impact on overall quality of life and mental health quality of life at 20 week follow up, however, this was not statistically significant. The intervention achieved reasonably high rates of engagement and retention, and participant feedback was generally positive.”

22-ii) Highlight unanswered new questions, suggest future research

A number of suggestions for future research are raised throughout the Discussion:
•“Further work is required to determine how to maintain physical activity behaviour change achieved by the Active Team app in the longer term”;
•“…the field of gamification for health behavior change is in its infancy and considerable further work is needed to explore its efficacy and optimal application”;
•“Further work is required to determine how to attract a diverse sample, and in particular, increase reach to low physical activity/low socioeconomic status users, who are likely to gain most benefit from a physical activity intervention. Furthermore, once effective intervention approaches have been devised, research focused on determining how to disseminate interventions on a mass scale, will be key. Insights offered by social marketers and traditional marketers are likely to be highly valuable in achieving these goals”;
•“Future iterations of the Active Team software will explore alternative recruitment structures, in order to draw on the positives of snowball recruitment, without the present limitations of the strict team structure.”

Other information

23) CONSORT: Registration number and name of trial registry

“…the study was registered with the Australian and New Zealand Clinical Trials Registry, protocol number: ACTRN12614000488606.”

24) CONSORT: Where the full trial protocol can be accessed, if available

For further details (beyond those which have been included in the Methods section), please contact the primary author (Carol Maher – Carol.Maher@unisa.edu.au).

25) CONSORT: Sources of funding and other support (such as supply of drugs), role of funders

Sources of funding are stated in the Acknowledgements section:
"Carol Maher is funded by a fellowship from the National Heart Foundation, and this project was supported by a South Australian Premier’s Research Fun Early Career Research Catalyst Grant."

X26-i) Comment on ethics committee approval

“Ethical approval for this randomized controlled trial was obtained from the University of South Australia Human Research Ethics Committee…”

x26-ii) Outline informed consent procedures

“Ethical approval for this randomized controlled trial was obtained from the University of South Australia Human Research Ethics Committee…Interested participants could access the app by typing “Active Team” into the Facebook search function, or by following a link included in the Facebook advertisement. The first page of the app was a welcome page, containing an information video and a detailed participant information sheet. Participants could then use the app to register interest in the study and complete baseline surveys.”

X26-iii) Safety and security procedures

Not mentioned specifically in the manuscript. The Active Team app contains a privacy policy page, which outlines how app users' information is stored and treated. Security of participant information collected via the Internet also outlined in the participant information sheet and online consent process.

X27-i) State the relation of the study team towards the system being evaluated

N/A - no conflicts of interest to declare; Active Team is a non-commercial application and was developed by the researchers with the assistance of Portal Australia.
